# Supplementary material for: Enhanced Photocatalytic Activity of Nonuniformly Nitrogen-Doped Nb2O5 by Prolonging the Lifetime of Photogenerated Holes
Source: Nanomaterials (Basel). 2022 May 16;12(10):1690. doi: 10.3390/nano12101690 (PMC9147847; doi:10.3390/nano12101690)
Supplement: Supplementary file 1 [file nanomaterials-12-01690-s001.zip › nanomaterials-1708835-supplementary.pdf]

Supplementary Materials

Enhanced Photocatalytic Activity of Nonuniformly Nitrogen-Doped Nb_2O_5 by Prolonging the Lifetime of Photogenerated Holes

Wei Guo, Chang Bo, Wenjing Li, Zhiying Feng, Erli Cong, Lijuan Yang and Libin Yang *

Tianjin Key Laboratory of Brine Chemical Engineering and Resource Eco-utilization, College of Chemical Engineering and Materials Science, Tianjin University of Science and Technology, Tianjin 300457, China; weiguo@mail.tust.edu.cn (W.G.); bochang@mail.tust.edu.cn (C.B.); lwj0606s@mail.tust.edu.cn (W.L.); zhing@mail.tust.edu.cn (Z.F.); cel@mail.tust.edu.cn (E.C.); yanglijuan@mail.tust.edu.cn (L.Y.)
* Correspondence: yanglibin@tust.edu.cn; Tel.: +86-0226060-1110

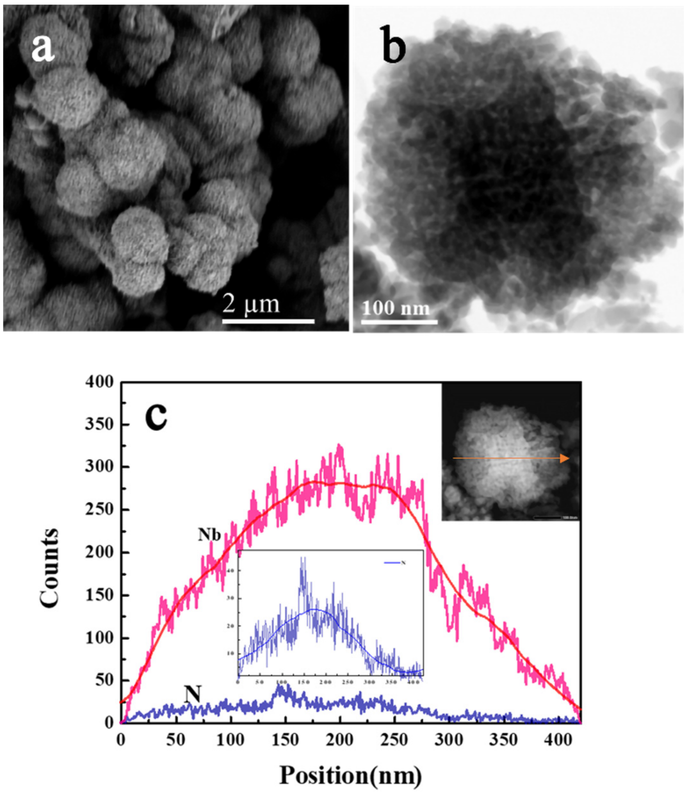

Figure S1. (**a**) SEM images, (**b**) TEM images and (**c**) EDS line scan of the 2@5@8@10NNbO.

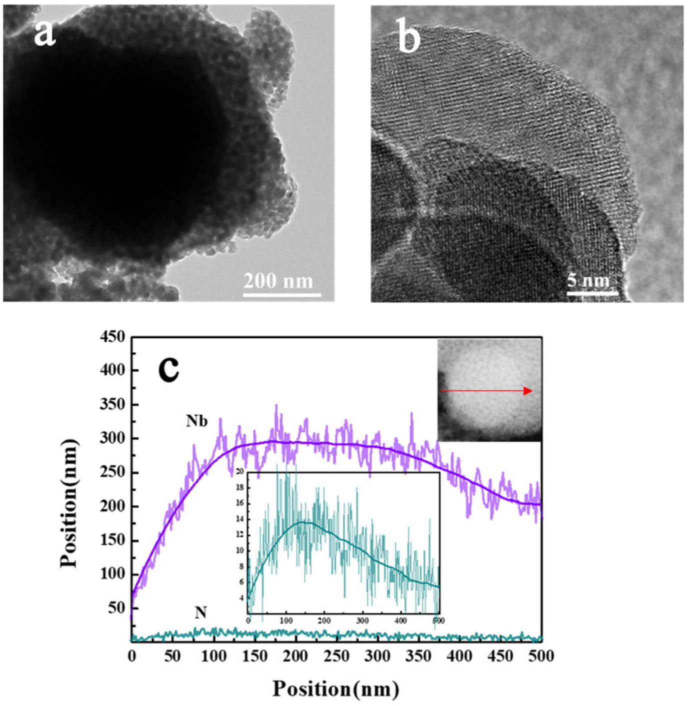

Figure S2. (**a**) SEM images, (**b**) TEM images and (**c**) EDS line scan of the Nb_2O_5@10NNbO.

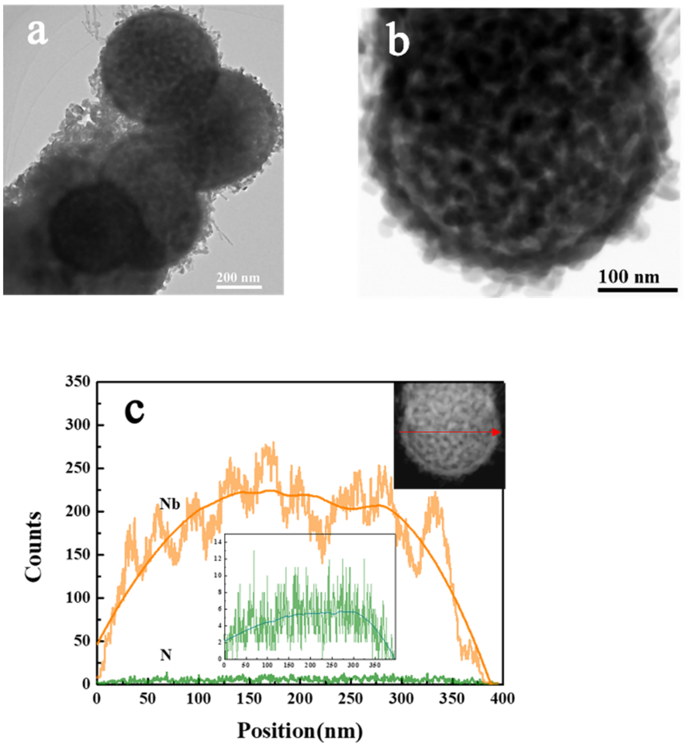

Figure S3. (**a**) SEM images, (**b**) TEM images and (**c**) EDS line scan of the 10NNbO@Nb_2O_5.

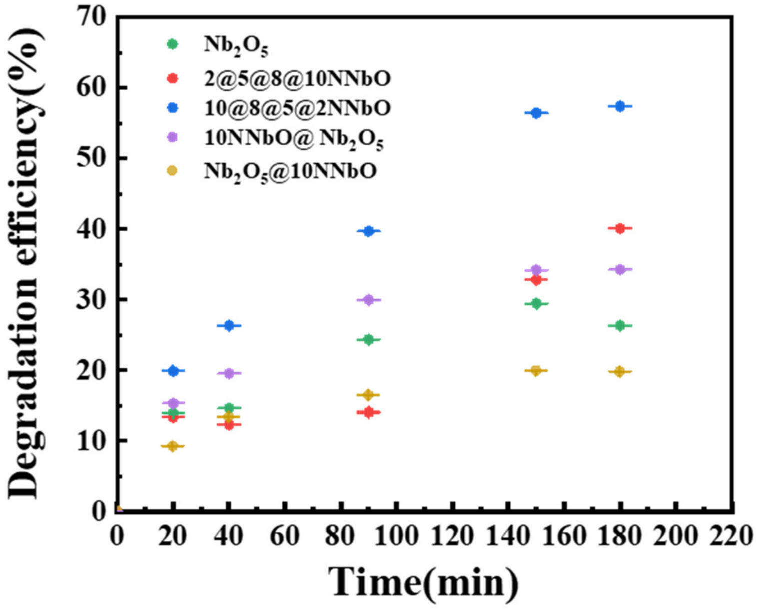

Figure S4. The degradation curves of photocatalytic materials varied with illumination time under UV radiation.
